# Connecting Masculinities to Men’s Illness Vulnerabilities and Resilience

**DOI:** 10.1177/10497323231198967

**Published:** 2023-10-30

**Authors:** John L. Oliffe

**Affiliations:** 1 School of Nursing, 8166 University of British Columbia, Vancouver, BC, Canada; 2 Department of Nursing, 2281 The University of Melbourne, Parkville, VIC, Australia

**Keywords:** men’s vulnerabilities, male resilience, men’s health and illness, masculinity, men’s mental health, case-study research

## Abstract

Men’s illness vulnerabilities and resilience are two predominant and regularly linked constructs in the masculinities and men’s health literature. There has been a steady stream of men’s strength-based vulnerabilities in the form of illness testimonials amid critiques that such disclosures are mere props for bolstering patriarchal power. The current article presents secondary analyses of case studies with four participants who took part in wide-ranging qualitative health studies to detail diverse connections between masculinities and men’s illness vulnerabilities and resilience. Prostate cancer–related vulnerabilities feature in the first case study where Arthur’s resilience for reclaiming his erectile function post-prostatectomy mobilizes an objection masculinity contesting his marginality. In the second case study, Chuck’s vulnerabilities are conceded as permanent flowing from his severe mental illness, a positionality situating resilience as obligatory for his survival. Here, Chuck embodies a resignate masculinity that accepts but works to manage the harms of his subordinate status. In the aftermath of his young son’s suicide, Jack laments that he did not model vulnerabilities. Resilience for understanding his loss influences a reimagined masculinity where Jack contemplates changes to gender norms for his and other men’s lives. Lastly, Sami replaces maladaptive actions for dousing vulnerabilities incurred through a partner-initiated separation with resilience for self-growth. Aspiring progress masculinity, Sami deconstructs his emotions and behaviors to positively change how he shows up as a man, father, and partner. The case studies reveal connections between objection, resignate, reimagined, and progress masculinities and men’s illness vulnerabilities and resilience to advance empirical, gender theory and methodological insights.

## Introduction

Men’s illness vulnerabilities and resilience are two predominant and regularly linked constructs in the masculinities and men’s health literature. Foremost, there has been a steady stream of and affirmation for men’s strength-based disclosures of their illness vulnerabilities in the form of public testimonials (Carless & Douglas, 2008; Hanna & Gough, 2016; Riessman, 2003). There have, however, also been claims of men’s patriarchy-driven motivations for faking vulnerabilities (McElroy, 2022) amid critiques that such disclosures are mere props for grand resilience narratives (Jordan & Chandler, 2019; Lomas, 2013). Interestingly, long-standing feminist critiques assert men’s stoicism, and resistance to feeling and/or expressing vulnerabilities are similarly driven by their desires to control others (McQueen, 2017). Clearly, debate abounds regarding the interpretations for what men do and do not share and embody in terms of their illness experiences and health practices. The current article reports findings drawn from secondary analyses of four case studies to detail diverse connections between masculinities and men’s illness vulnerabilities and resilience.

### Vulnerabilities, Resilience, and Masculinities in Men’s Health

By definition, vulnerabilities typically comprise a situation where shame, fear, stigma, and/or marginalization occur (Baiasu, 2020). In the men’s health literature, there is wide-ranging valence for how vulnerabilities have been positioned and depicted. For example, early on, Sabo and Gordon (1995) suggested it was men’s masculine risk-taking that left them *vulnerable* to injury and preventable diseases. In the COVID-19 context, de Boise (2021) explained men’s risky behaviors (i.e., reticence for wearing masks and/or resistance to being vaccinated) as countering visible unmanly *vulnerable states*. Young men have been reported to draw from *discourses of vulnerability* to justify accessing primary healthcare services (Jeffries & Grogan, 2012). After prostatectomy for prostate cancer, men’s *vulnerability and caution* was used to label their conservative (and wise) recovery enhancing approaches to physical activity (Gannon et al., 2010). Health inequities have also been used to lobby tailored health promotion interventions for *vulnerable* male sub-groups including Indigenous (Efimoff et al., 2021) and sexual minority men (Lee et al., 2017). Taken together, men’s illness experiences and disease disparities can be understood as shaping and being shaped by context-specific individual and sub-population vulnerabilities. Linking resilience, Trundle et al. (2018) differentiated some vulnerabilities as harmful and others as offering life affirming opportunities. Likewise, Baiasu (2020) claimed transformative gains could be made by harnessing the resilience to adapt and effectively deal with vulnerabilities. It is in this positive response frame that men’s resilience for managing and overcoming illness vulnerabilities emerged as strength-based projects (Douglas, 2003; Fox, 2003; Reeve, 1999, 2004). Similarly, health inequities naming specific male sub-groups as comparatively *more vulnerable* to poor health outcomes are increasingly critiqued as further marginalizing those collectives, which in turn has led to petitions for acknowledging and helping to mobilize existing community-based resilience and assets (Darroch et al., 2021; Pruden et al., 2021).

Operating across men’s illness vulnerabilities and resilience, masculinities (Connell, 2005) have prevailed as the most commonly used gender theory and explanatory framework. Connell’s (2005) plurality of masculinities describes men’s characteristics and performativities in relation to hegemonic masculinity. Specifically, delineated are men-in-relation as complicit in sustaining, as well as marginalized, subordinate, and/or protesting hegemonic masculinities (Connell & Messerschmidt, 2005). The early masculinities and men’s health work reported illness vulnerabilities as synonymous with weakness (Courtenay, 2000; O’Brien et al., 2005). More recently, disclosing illness vulnerabilities has emerged as strength-based, a courageous practice for norming minority and debility states, rewriting manly values, and easing the hold of restrictive masculine ideals (Hanna & Gough, 2016). As Barounis (2019) reports in her *Vulnerable Constitutions* volume, the visibility of sexual minority men and those experiencing physical damage has given rise to new models of masculinity. Within this context, the drive for authenticity has infused men’s illness identity disclosures with promissory notes for de-stigmatizing and emancipating all men to openly talk about (rather than conceal) their vulnerabilities.

There have, however, also been suggestions that men’s illness vulnerabilities are a somewhat saturated market—with the aforementioned emancipatory potentials diluted by the sheer volume, over-exposure, and suspect motivations for such public releases. At the extreme, a *New York Times* letter titled *Toxic masculinity is now petulant vulnerability* suggested men were feigning emotional fragility to retain power and dominance (McElroy, 2022). Here, McElroy (2022) argued that men’s fake vulnerabilities were manipulative in continuing the patriarchal project. On a continuum of critiques, there were also assertions that men’s vulnerability disclosures are mere props for their grand resilience narratives (Jordan & Chandler, 2019; Lomas, 2013). That is, the revered manly “good fight” (Halpin et al., 2009) and gritty self-reliance narratives (Hutchinson & Kleiber, 2000) have been questioned as reinforcing hegemonic masculinities where men courageously contest and/or conquer their vulnerabilities. This masculine discourse even (and perhaps especially) prevails in memoriam, when life-ending acute injury and/or hard-fought chronic illness battles are lost by brave resilient men (Douglas, 2003; Reeve, 1999, 2004).

In sum, there is much debate about the social and structural gender influences on men’s disclosures of their illness vulnerabilities and resilience. The current article presents secondary analyses of case studies with four participants who took part in wide-ranging qualitative health studies. The case studies reveal connections between objection, resignate, reimagined, and progress masculinities and men’s illness vulnerabilities and resilience to advance empirical, gender theory and methodological insights.

## Methods

Thorne’s (1994) secondary analysis framework for qualitative data informed the current study design, and selection of case studies from four wide-ranging men’s health projects to explore connections between masculinities and men’s illness vulnerabilities and resilience. Purposefully acting on emergent lines of inquiry, secondary analyses also guided the inductively derived research question for the current study (Thorne, 2013). A case study approach was used to develop depth and nuanced gender analyses specific to each participant (Baškarada, 2014). As Flyvbjerg (2006) suggests, context-dependent knowledge drawn from case studies is critically important to advancing the social sciences through illuminating actors and constructs within specific health-related topics. Writing myself into the data collection reflects *being there* in co-constructing the data and positively responds to recommendations that reflexivity and creative non-fiction writing best meets contemporary expectations of qualitative research (Caulley, 2008).

## Data Collection

Ethics approval for secondary analyses was granted for each of the four projects from which the case studies were drawn. The four case studies comprise men with diagnosed, formally treated illnesses and participant’s experiencing threats to mental health invoked by unforeseen life transitions. Diverse cases were included to ensure variation in exploring how masculinities and illness vulnerabilities and resilience connect in men’s lives.

The first case study featured Arthur, a 46-year-old man who underwent a prostatectomy for prostate cancer. Arthur participated in the author’s 2001 PhD research, an ethnographic study of heterosexual Australian men living with prostate cancer (Oliffe, 2005). Chuck, a 51-year-old Canadian man who experienced severe mental illness and suicidality, features in case study 2. This 2014 interview was conducted as part of a study focused on de-stigmatizing Canadian men’s mental illness and suicidality (Oliffe et al., 2021a). Prostate cancer, as the most commonly diagnosed male cancer, and connections between mental illness and men’s high suicide rates influenced the inclusion of Arthur and Chuck’s established illness case studies. Jack, a 52-year-old man based in rural Alberta, Canada, took part in a 2015 interview study addressing male suicide bereavement (Oliffe et al., 2018). He had recently lost his 16-year-old son to suicide. The final case study featured Sami, a 47-year-old Australian-based father whose partner initiated the break-up of their 10-year relationship. In line with COVID-19 restrictions, Sami’s 2022 interview took place via Zoom (Oliffe et al., 2021b) and was part of a study focused on men’s mental health in distressed and disrupted intimate partner relationships (Oliffe et al., 2022). Well-established linkages between men’s life transitions (i.e., bereavement and relationship break-ups) and mental health risks prompted the inclusion of Jack’s and Sami’s case studies.

## Data Analysis

The author was familiar with the participant interviews and had previously analyzed these data in addressing the primary research questions for each of the respective studies. In re-listening to the interviews and re-reading the transcripts, the feasibility of the data illuminating men’s vulnerabilities and resilience were positively evaluated, and the secondary research question *What are the connections between masculinities and men’s illness vulnerabilities and resilience?* was developed. A descriptive case study approach was used to detail different characteristics of masculinity and men’s illness vulnerabilities and resilience. In reading each case study numerous times, jottings were made to note key events, perspectives, and storylines, and preliminary interpretations of the interview and relevant data were documented (Baškarada, 2014). As a unit of analysis, each case study was summarized, and potential illustrative quotes for detailing the findings were coded.

The case studies’ descriptive labels preempt the findings regarding *their* connectivity between masculinities, vulnerabilities, and resilience. The findings reveal men’s embodied vulnerabilities and how resilience served to contest, accept, contemplate, and enrich their masculine states. Here, the four case study findings differentially comprise objection, resignate, reimagined, and progress masculinities. A classificatory approach (Gerring, 2004) was used to augment rather than test Connell’s (2005) masculinity theory. Though presented separately, the case study findings should be understood as process states that may overlap for many men, including the four current participants.

## Resilience for Contesting Vulnerabilities Through Objection Masculinity

It is 2001, and I am in the leafy Melbourne suburb of Hawthorn meeting with Arthur, a 46-year-old man who has been treated with prostatectomy for prostate cancer. Arthur’s terrace home features a luxury sofa on which I am perched, tape-recorder in hand, ever-ready to capture his story. We are close in age, and it is as though Arthur needs a mate—the rare sort who might listen and understand—or perhaps just listen to what is *really* happening for him. Delaying the interview, Arthur asks if we can walk down to the nearby school to pick up his son. We chat all things football as we stroll ahead of a young boy running into Arthur’s outstretched arms just inside the school gates. He asks, “Daddy, who is that?” “Mitch, this is John—he’s a friend of daddy’s.” Mitch is 5 years old, and he does not know that daddy has prostate cancer. We arrive back at the house, and Emma (Arthur’s wife) takes Mitch by the hand, as they are soon heading off to grandma’s home as per their Thursday ritual. Emma quizzically looks at me prompting Arthur to repeat “this is John” elaborating “he is a researcher and I am going to chat with him about the PC.” Coded, PC denotes prostate cancer (not personal computer)—and Emma, catching her own eye-roll mid orbit, bypasses a formal greeting (or indeed another word) and is gone, the only trace a mother–son silhouette exiting the front door. Arthur joins me on the luxury sofa, and shoulder to shoulder with comfortable distance between us, the formal interview finally begins.

The interview with Arthur started long before the recorded conversation to reveal some important insights. It is fair to say that Arthur assessed the worth and safety of talking openly with me about his prostate cancer. There are, of course, vulnerabilities for men talking about illness, and their self-disclosures depend on who is being spoken to, and to what end. In addition to determining my purpose and fit, Arthur initiated the side-by-side seating arrangement, avoiding any direct line of sight and, by extension, the visibility of wayward emotions that might escape to derail his narrative. The protection of Mitch in coding prostate cancer as PC also limited the young boy’s exposures to illness vulnerabilities and normed Arthur’s protective stoicism as a selfless, strength-based manly practice. I also learn that Emma’s reaction and exit reflected her growing frustrations, which according to Arthur elevated whenever he belabored his primary prostate cancer–induced worry—erectile dysfunction. Emma had lobbied Arthur to refocus on his job and family, especially given the positive post-surgery news that he was free of prostate cancer. Not that Arthur’s erectile dysfunction vulnerabilities weren’t valid, Emma just needed him to fully rejoin their pre-prostate cancer lives. The relational nature (and temporal dimensions) of illness vulnerabilities and resilience was evident in Arthur’s and Emma’s differing priorities, and some new and unexpected challenges to their partnership had emerged.

Arthur began the interview by framing his erectile dysfunction (not prostate cancer) as *the* omnipresent vulnerability drawing his resilience for contesting that marginalizing state. Rightly situating himself as young to be diagnosed with prostate cancer, Arthur’s worry about dying ended post-surgery. But spoiling that positive outcome, Arthur grieved for his masculine-self, the lover he had been, and the sexual pleasures he’d known and shared with Emma. He also somewhat grappled with being young enough to *still* have a strong libido but looking needy or parody-like in his middle-age pursuit of restorative erectile treatment[s]. Objecting the latter, Arthur argued against feminist views that “you shouldn’t have Viagra available because they [men] should find other ways to enjoy their sexuality.” He continued, “a lot of women like penetrative sex” so “people who haven’t got the problem [erectile dysfunction] shouldn’t make judgments about other people’s sexuality.” Arthur’s disquiet was also compounded by Emma because “even though she will say ‘I can be pleased in other ways’…there is no doubt about it, she likes the penis penetration” and “a lot of relationships have broken down it seems when the sex stops.” Arthur’s objection masculinity was characterized by a refusal to accept his erectile dysfunction, even as a concession for being free of prostate cancer. Rather, he relentlessly pursued remedies for that debility state. In sum, he railed against the loss of an important part of his masculine identity, threats to his relationship with Emma, and social pressures that he should learn to live with his erectile dysfunction. Beginning with the vacuum erection device (VED), Arthur wryly explained the mechanics of that “unpleasant ordeal” wherein he placed his flaccid penis in a plastic tube and hand-pumped the external bulb to create pressure inside the cylinder to summon blood for an erection:

It made my penis get fat at the base but not exactly grow. It had these inch notches marked on it [the VED] … and there was still a good two inches of space for me to grow into … I could get an erection out of it but not like I used to get … it was just really painful … it [Arthur’s penis] was pushing right against the tube you could see the skin pushing up like someone’s face against a window.

Arthur subsequently tried Viagra; however, that “did not always work” and he got side effects of “headaches … sinus … indigestion” and “could not sleep after using it.” Thereafter, he decided to try “the injection,” explaining the first one was administered at the clinic to teach him how to inject himself at home in the future. Arthur got an erection following the injection at the clinic and quickly made his way home but “unfortunately I couldn’t use it [the erection] because Emma had an appointment in the afternoon and … I had to go to a meeting that night.” Arthur tried the injections twice more, but “the pain was murder and lasted for as long as the erection. Nearly four hours.” Ineffectual and invoking dismal side effects, Arthur conceded the three treatment misses amid assuring me of his ongoing resilience for finding *the* cure for his erectile dysfunction.

Arthur and I shake hands, and I depart after our two-hour interview. As is often the case, Arthur and I never meet again. The objection masculinity contesting his erectile dysfunction vulnerabilities had been disclosed to teach me and all who subsequently read him. Contrasting Arthur’s objection masculinity and resilience for ending his marginality, the second case study featuring Chuck highlighted resignate masculinity as conceding a subordinate state invoked by mental illness vulnerabilities.

## Conceding Vulnerabilities With Resignate Masculinity to Position Resilience as Survival

It is 2014 when I join Chuck, a 51-year-old man who has long experienced severe mental illness, to talk about his depression and suicidality. Snow-capped mountains encasing Vancouver, Canada, are backdrop to the university office where Chuck’s grim account fills our two-and-a-half-hour interview. Faded shoulder-length hair unevenly falls on Chuck’s frayed blue floral shirt collar, melding into a salt and pepper stubble which frames his drawn weathered face and locking gaze. Chuck is immediately likeable but worrisome, friendly but distant, expert for what he knows and feels, yet entirely uncertain about who, and the feasibility for how he *really* is. He begins our interview by telling me he comes “from a family with a long history of mood disorders—depression, clinical depression as well as bipolar” ahead of disclosing that he has been depressed and had suicidal thoughts for the last 16 years. In all that unfolds, Chuck’s vulnerabilities flow to and from his mental illness, wherein he understands his depression and suicidality as trait-based permanent fixtures that render him forever a victim to life’s negative events:

It’s hard to describe the unhappiness, it’s not the kind of unhappiness that is environmentally influenced, it’s not like an unhappy marriage or an unhappy job, or something that I can put my finger on and say, “That is the source of my unhappiness.” Um, it was much more innate to who I felt I was, or who I feel I am.

In essence, life was cruelly happening to Chuck to the extent that he conceded his core vulnerability (mental illness) in positioning his resilience as life-sustaining labor to withstand significant disadvantage and damage. He recounted being diagnosed with depression, and opting for anti-depressant medications, ahead of lamenting that seeking and receiving help did not stem his stream of life losses:

In my mid to late 30s I was going through a period of time where I was trying different anti-depressants with little success, I had a very good job, I was a manager, and admittedly my behavior had become a little bit erratic, my impulse control had been somewhat compromised. I don’t know whether or not that was a side-effect of one of the meds [medications] I was taking … and uh, I got into bit of a verbal disagreement with my supervisor … and she accused me of being a potentially violent or dangerous person, and I was essentially forced to resign.

In the aftermath of relinquishing his job, underemployment followed, and Chuck’s routine, purpose, and provider identity wallowed and waned. These vulnerabilities layered Chuck’s life manifesting a resignate masculinity characterized by an acceptance of the permanency of *his* mental illness and subordinate state. Chuck conceded his lack of purchase for hegemonic masculinities. Indeed, numerous cause–effect scenarios feeding his hopelessness and self-assigned subordinate place were offered to accede his positionality. The interview was without refrain, reprise, or a glimmer of hope for improvement or recovery. Rather, Chuck’s narrative built to the disclosure that he had been the victim of child abuse:

Age 42 I literally got out of bed one day, and was hit with a hammer around abuse that I had suffered over a 2-year period when I was 8 years old, from 8 to 10. It was a series of events that I had very neatly packaged up into a box and I’d put it up on the back shelf in my brain, and I bumped my head one day and the box fell off, and all the contents just spilled out—it was acute over the span of a few days, I all of a sudden went from being this normal suburban reliable, responsible husband, father, all the rest, and banished myself to living on the Downtown Eastside [an area of Vancouver known for its disproportionately high levels of poverty, drug use, homelessness, crime, mental illness and sex work] without my family knowing where I was, I left with the clothes on my back with the sole purpose of destroying myself.

Chuck explained that it was not until the fifth year of regularly meeting with his psychiatrist that he talked about the abuse he had experienced as a child. There are, of course, vulnerabilities for speaking of, and to such traumas, with the abuse of boys being amongst the most silenced, stigmatized, and shaming wrongs to disclose (and right). However, Chuck bracketed those traumas separating significant childhood injuries from his mental illness challenges. Instead, his resilience for staying in therapy and working to survive a lifetime of vulnerabilities featured within a resignate masculinity that accepted his subordinate state and status.

Chuck’s interview was heavy, and a challenge to having a clinical background when interviewing men about their health is holding in abeyance *your* direct professional help. Here lay some clinician-researcher vulnerabilities and resilience, and perhaps Chuck sensed that in his unsolicited close to our interview:

You gave me a wonderful opportunity, and—sorry, I don’t mean to sound patronizing, but your questions—your follow-up questions in particular were really good, because you got me to think about things that I hadn’t considered, so, you prompted some other thoughts for me. So, that was worthwhile for me, certainly … It’s just that sense of closure, sometimes they’re just little things, and sometimes they’re big things, but all the little things add up to something worthwhile, so thank you for that.

Chuck’s comments confirmed illness vulnerabilities don’t have to be remedied directly; instead, there is value in talking and being heard, and further processing thoughts and events. Also, the therapeutic value of qualitative interviews was evident in that Chuck used the forum to authentically (and anonymously) speak to *his* vulnerabilities and resilience.

Chuck’s resilience to withstand his mental illness vulnerabilities featured within a resignate masculinity that accepted his misfortunes as subordinating and separating him from healthy uninjured men. While these vulnerabilities layered and stayed, they also drew Chuck’s resilience work to survive an ill-fitted (and unfair) life. The third case study highlights the reach of suicide, wherein Jack’s vulnerabilities and resilience conjure a reimagined masculinity in the aftermath of losing his son to suicide.

## Resilience for Norming Vulnerabilities in Contemplating a Reimagined Masculinity

Jack, a 52-year-old man lived on a farm with his family in rural Alberta, Canada, but worked in the city of Edmonton, a 90-minute drive from his family home. A research assistant interviewed Jack, but I did meet him late 2015 at an event aimed at de-stigmatizing men’s mental illness and preventing male suicide. I had read Jack’s interview before meeting him, and the rawness, reserve, and rationality of that transcript prevailed during our lengthy in-person chat at the community event. Jack was personable, insightful, welcoming, and warm, and I am forever grateful for his generosity amid fragilities ablaze in candidly telling us about losing his 16-year-old son, Wes, to suicide:

Wes was finishing up his school year, grade 11. It was June 26 a Tuesday, I’d left work … and I knew he had his last exam that day … so on the way to the mall to get my haircut I phoned him, he didn’t pick up. It’s not that unusual, it’s quarter to five, so I phoned Hanna [wife] just for somebody to chat with while I’m driving, she said, “Oh didn’t Wes phone you?” … I said, “No, he hadn’t.” Anyways, she told me a story that on this exam that day he’d been one of a handful of students that had been cheating, and they’d all got caught. He was gonna have to rewrite this test and Hanna told Wes that he needed to phone me about it. I sent him a text to call me and Hanna said she was gonna go home. I went and I got my haircut and I’m walkin’ out and my phone rings and I can see it’s my home number and I answer it and I can’t … it’s the most awful noise coming out of this phone and I can’t imagine what it is to start with and after a moment I’d figure out that it’s Hanna, just screaming. I don’t know what’s happened, but I know it’s the worst thing ever and then she stops and starts sobbing and I still can’t understand what she’s saying and eventually she tells me that Wes hung himself. And she’s there by herself. I’m an hour and a half away.

Wes had left a note explaining he had been depressed for some time; he also expressed his love for the family amid apologizing for ending his life. He wrote, “try to see it as my pain ending” conceding “I feel terrible for being a disappointment to you dad.” As you might expect, Jack and his family endured considerable complicated grief. Much of this centered around the discordance between their read of Wes’ seemingly high energy levels with the latent (and languished) information contained in his letter that he had been experiencing severe depression for some time. An endless stream of moments and memories played for Jack replays of what might have been warning signs to prompt his life-saving actions to prevent that tragic loss.

Jack’s vulnerabilities also featured as uncertainties about his own influence on Wes’ concealment of his depression and his son’s stoicism for bearing what ultimately grew to be intolerable pain:

I’ve just thought since, I didn’t think about all the stuff that I could have taught him. If there was one thing … I would handle differently, it would be about vulnerability for myself—I think if we tell all our kids the good stuff, the great stuff, then that’s what they see. Every time they see you as an adult you’ve kind of got your stuff together … you don’t tell them all the mistakes you made; you don’t tell them the stupid things I did … I think he had a perception that perhaps I didn’t have these feelings and vulnerabilities and that I wasn’t scared of things, and I didn’t hurt. And because of that he did think that he didn’t measure up, and quite the opposite was probably true.

Jack lamented covering up his own vulnerabilities and the influence on Wes’ silence and avoidance for being seen as weak for needing some help to ease his depression. The small-town rigid masculine norms were also deeply implicated, wherein men were idealized as stoic and strong. At the event where I met Jack, some townsfolk asserted that such hegemonic masculinities had influenced the recent suicides of six young local men (including Wes). Jack had tried to orientate Wes to rural and urban worlds to placate some of those small-town pressures, but in the aftermath of his son’s suicide, he reimagined masculinity wherein distressed men could be seen, heard, and helped:

It has made me a better person, if you were judging people, you know, it softened me … One of my best friends, his son is struggling with alcoholism due to depression, and I would do anything to help him ... I think for boys and men those voices [vulnerabilities] will be quiet. So, if you hear a whisper that he’s struggling with this, he might not say “I’m struggling with this,” he might say you know, “math sucks.” If you were hypersensitive to that … listen … don’t talk, don’t advise, just very intentional listening is needed.

Jack suggested he was more aware of, compassionate about, and sensitized to the vulnerabilities and struggles young men faced, and his reimagined masculinity was envisioned as less restrictive for himself and for the small-town community more broadly. His contemplations for these changes did, however, rely on some of the hegemonic masculinities he had hoped to disrupt:

I’ve had lots of talks with people, and the support, the counseling was all about us and how I’m coping with things and managing things … I also have some good friends that I’ve been able to talk to ... slowly you get back up right … you decide you’re going to get out of bed that day and you make decisions for yourself and what you’re going to do. And, you get going and you keep going.

Jack’s narrative confirmed the push and pull of vulnerabilities and resilience in his own life and bereavement. Underscoring the harms of concealing vulnerabilities, Jack’s reimagined masculinity was a contemplative state—mired in deciphering how to better read and redress risky masculine norms to garner positive life-saving actions.

Jack’s vulnerabilities were fact—visibly part of who he now was and his resilience for finding meaning and change for himself, other men, and the world more broadly. While Jack contemplated reimagined masculinities, the fourth case study featuring Sami revealed progress masculinity—a strategic action orientation for working with his vulnerabilities to be a better man, father, and partner.

## Vulnerabilities Levering Resilience Work for Progress Masculinity

In a study of men’s mental health and intimate partner relationship break-ups, I interviewed Sami via Zoom in 2022. He popped up on the screen with a wry smile lauding the benefits he anticipated getting by talking with me from his home in Melbourne, Australia. Sami was a 47-year-old father, who from the outset spoke about how his progress masculinity, comprising intentional self-work, was catalyzed by his partner (Sue) initiating the break-up of their 10-year relationship. Decisive but processing, and craving objectivity amid trying to untangle his role in the demise of the partnership, Sami’s vulnerabilities rallied his reflexivity for all things, ranging the courtship through the distress in and after the relationship ended:

I think really what was attracting to me was her vulnerability and because I was in the mindset and the habit of saving people, especially women, it was just “hey that’s what I do right,” I’m saving women who need saving … just saving damsels in distress.

In response to my follow-up question asking what Sami thought had initially attracted Sue to him, he said, “I guess it was that protector, that someone who can fix things.” Catching himself, Sami stopped mid-sentence conceding, “I know there is work to be done there” in referencing his need to be less presumptuous about the motivations for and fit of intimate partners. Sami also mapped the early distress signs leading up to the end of the partnership, “the cracks started to show with the physical intimacy, with sex just becoming less, having less and less of it.” Uncertain about how to bridge their ever-increasing distance, Sami focused on staying in the relationship, especially in light of the fact they had just had a baby, even after Sue ended the partnership:

She just said “look, I can’t do it anymore; I don’t feel anything. I feel like this is not working. Basically, I want out.” So, that was the reality suddenly that I needed to deal with, so I went into what I did best at that time, which is denial and excuses and trying to find a way we can work on it, and maybe we can do this and that, and the answer was “no, I don’t want to work on it, there is nothing to work on, that’s it.” It took me two years of living in denial, just this limbo state of not here, not there.

Sami and Sue slept in separate bedrooms in what was effectively a shared house and co-parenting arrangement for those two years. Retrospectively, Sami suggested that during this period:

I was denying my emotions and denying myself. It was just shoving things under the rug … I was feeling confused, I was lost, I was feeling sad, a deep sadness. I was really feeling a lot of shame involved and a lot of fear … I went back to smoking pot … and when the shit hit the fan, I started smoking [cigarettes] again. I was drinking every day.

Sami’s vulnerabilities grew with the ineffectual dousing of all that he felt about the relationship and the break-up. Recognizing his pain and crisis, Sami spoke to *his* vulnerabilities levering *his* resilience work for progress masculinity:

I’m desperate, I have to do it, something has to change … the relationship with myself … my separate started when I separated from myself. It wasn’t her [Sue], it wasn’t the relationship, it was me; I needed to work on myself and get in better touch with myself, to understand myself better, to understand my shadows better, to understand my habits. “Why do I do what I do?” “Is it healthy for me?” “How do I feel being in touch with my emotions?” All that self-development, self-growth, whatever you want to call it, that’s where it starts.

Deep on introspection, Sami reframed his vulnerabilities as opportunities to resiliently work on himself. Evident also in his progress masculinity was an emphasis on undoing some masculinities to build something better:

Men are not taught emotional tools; that’s actually bred out of them. So, what I’ve heard is don’t show emotions, vulnerability equals weakness, don’t be a girl, men don’t cry. So, that means that when I had an emotion crisis, I’m left without a toolbox; I don’t know how to deal with this … what is important to me, and the word that came up was growth … I just want to be heard, I just want to be honest, what you said triggered me and I feel this, this and that. I’m not asking for you to change anything. I just want to tell you, this is what I felt, and it’s okay, I’m responsible for my own feelings, I’m going to work on it, thank you, that’s it.

Sami relinquished some restrictive hegemonic masculinities to which he had aligned, to fully engage, express, and take responsibility for what he felt. Addressing his withdrawal, blame on Sue for what he felt, and the denial of the relationship ending, Sami’s progress masculinity was contingent on his resilience for understanding (and addressing) his vulnerabilities. Referencing a new relationship, Sami positioned this self-work as ongoing, explaining his strategies for dealing with his partner’s decision to go out to dinner with her neighbor:

Okay, I’m going to sit with it and I’m going to figure it out; what am I feeling. I’m feeling, jealous. I’m feeling a bit of fear maybe because maybe she’ll go and doesn’t want to be with me anymore, that’s a lot about ego … It’s about providing a safe space for this … vulnerability.

With conscious and committed work, Sami’s vulnerabilities were deconstructed and worked on to reconcile and better embody how he wanted to feel, be, and show up in the relationship. Positioned as a liberating praxis, Sami also understood this vulnerability work as demanding his progress masculinity work lifelong.

## Discussion and Conclusion

The current study offers empirical, gender theory and methodological insights to advance long-standing debates about men’s illness vulnerabilities and resilience. While acknowledging that there are men who will forever deny and conceal their vulnerabilities, the current study, by sharing participants’ forthright accounts, illuminates diverse and powerful connections between masculinities, vulnerabilities, and resilience. Contrasting Arthur’s commitment to contesting his marginality with Chuck’s acceptance of his subordinate state, and differentiating Jack’s contemplative changes from Sami’s in-progress self-work, wide-ranging contexts and complexities were shared in each of the case studies. Simply put, illness vulnerabilities levered a plurality of objection, resignate, reimagined, and progress masculinities, varied process states that in and of themselves beckon equity, diversity, and inclusion frames to comprehend all that constitutes and counts as men’s vulnerabilities and resilience. In what follows, each case study is discussed separately, ahead of offering some gender theory and methodological viewpoints based on completing the current research and article.

Arthur’s case study offers a poignant example of how acute loss can disrupt masculine identities, roles, and relations to invoke significant and oftentimes unanticipated vulnerabilities. Arthur’s resilience for remedying his erectile dysfunction, while positioned as restorative for his and Emma’s intimacy, primarily contested *his* marginality. Asserting *his* need and rights to re-establish *his* erectility and sexual prowess, Arthur’s objection masculinity reflected reliance on erection, penetration, and climax—sexual performativity synonymous with hegemonic masculinities (Connell & Messerschmidt, 2005). Men’s specific vulnerabilities can draw judgments (and defenses) about the gendered motivations for addressing some deficits (Lomas, 2013). More generally, Arthur’s resilience for contesting his marginalizing loss was in line with men’s most often-told illness vulnerability story, wherein vulnerabilities summon men’s masculine strength for contesting and ideally combating marginality, as previously reported by Jordan and Chandler (2019). Naming this, objection masculinity seems especially likely in response to acute loss. Of course, when those losses sustain (or worsen), as was the case for Chuck, resignate masculinity can emerge.

Chuck’s resignate masculinity accepted his mental illness vulnerabilities but prioritized withstanding the unending challenges that flowed from that debility state. Resilience as requisite for staying alive was triaged to manage those ever-present, layering mental illness vulnerabilities. While Connell’s (2005) work has positioned hegemonic masculinities as the construct by which complicit, subordinate, marginalized, and protest masculinities are assigned, Chuck’s resignate masculinity conceded and operated within the subordinate masculinities’ arena. That Chuck’s chronic vulnerabilities demanded his life-saving resilience work raises concerns about the potential for his steely resolve to erode over time and tilt him toward self-harm. As Trundle et al. (2018) differentiate some vulnerabilities as harmful, and there is significant suicide risk for men experiencing chronic, severe mental illness and suicidality challenges (Oliffe et al., 2021). Characterizing resignate masculinity also was Chuck’s a ambivalence for hegemonic masculinities—ideals wholly known to him as out of reach. Instead, Chuck’s life struggles manifested a survival mode for living with, rather than a remedy-based resilience to correct, his subordinate state. Differentiating Chuck’s resignate masculinity, Jack’s reimagined masculinity, though contemplative, was a more hopeful state.

Jack’s reimagined masculinity responded to his and other men’s vulnerabilities to push his resilience work for making sense of, finding meaning through, and preventing the devastating losses that can flow from men concealing their distress. The contemplation for how and where to embark on this reimagined masculinity project predominated, and the complexities of Jack’s grief likely limited his actions for self and structural change. For example, while some of Jack’s vulnerabilities featured in the interview, his resilience also included hiding those states as a means to being strong and supportive for his bereaved family. In line with Chandler’s (2021) assertion that dominant discourses of masculinity can block men’s actions for disrupting hegemonies, one of Jack’s other compromises was to better read and discreetly respond to (rather than disrupt) men’s masculine norms for concealing their mental illness vulnerabilities. In essence, while Jack’s reimagined masculinity critiqued some harms of hegemonic masculinity, his narrations for change were contemplative and at times reflected the gendered practices he critiqued. While Jack recognized the need for change (reimagined masculinity), Sami’s progress masculinity was characterized by decisive actions and self-work.

Sami’s progress masculinity emerged from an often-told crisis narrative whereby avenues (e.g., substance use and concealment of emotions) synonymous with hegemonic masculinities increased vulnerabilities to intolerable levels. Here, Sami’s rock bottom levered his resilience for abandoning denial and self-medicating practices in favor of purposefully forging a new and improved masculine-self. Incorporating some redemptive elements, Sami’s self-work was a strength-based, asset-building project, both requisite and mandatory to him being a better partner, father, and man. As Baiasu (2020) highlights, some vulnerabilities, as evident in Sami’s case study, offer positive life-changing opportunities. Sami’s interview also highlighted his resilience for vigilant introspection and labor to sustain a progress masculinity. Indeed, Sami’s lifelong commitment to deconstruct his vulnerabilities to adjust his behaviors equally disrupted (e.g., introspection and emotion work) and relied (e.g., strength and self-reliance) on hegemonic masculinities.

These case study findings should be acknowledged as participants’ anonymous self-disclosures in qualitative interviews, with consideration to how formal research analyses might offer distinctly different purviews than the social media critiques of men who publicly share their illness vulnerabilities. This is not to espouse men’s private and public vulnerability disclosures as separate or disconnected. Inversely, these narratives are intricately tied, as Chandler (2021) highlights, dominant discourses of masculinity influence men’s talk (and silences) about their illness challenges. Affirming the connectivity between men’s private and public disclosures of illness vulnerabilities, I suggest that McElroy’s (2022) *New York Times* letter—*Toxic masculinity is now petulant vulnerability*— perpetuates (perhaps inadvertently) the patriarchal project critiqued therein, with accusations that men “feign emotional fragility as a means of retaining power.” Specifically, shaming men’s public vulnerability disclosures as inauthentic risks renewed silences and inequities—especially for men living in marginalizing conditions and/or chronic subordinate masculine states. In addition, there is little refuge for men who comply to stoically conceal their illness vulnerabilities because they can be subject to feminist critiques asserting that such strong silent type embodiments are similarly driven by patriarchal power and control over others (McQueen, 2017). My point here is to say that dominant discourses of masculinity influence men’s public disclosures of their illness vulnerabilities—with flow on effects for what is normed and privately shared (and endured). Bearing this in mind, it is important to respect men’s lived experiences as their subjectivities, and reflecting diverse agency and structure entanglements. So, while McElroy (2022) might be rightfully suspicious in doubting some men’s motivations for publicly disclosing *their* vulnerabilities, we need to thoughtfully consider how, in a post-truth world (Sismondo, 2017), vulnerabilities are taken up in relationships, health care, and society more broadly.

Regarding gender theory, while Connell’s (2005) masculinities framework has long featured in men’s health research, offering objection, resignate, reimagined, and progress masculinities in the current study offers some important process states for mapping men’s gendered performativity and practices. Specifically, illness vulnerabilities and resilience entwined as dynamic within and across Connell’s (2005) marginalized and subordinate masculine categories to confirm a continuum of men’s reliance on and rejections of hegemonic masculinities (Connell & Messerschmidt, 2005). The gender theory contribution in the current study is simply to remind us that the categories in Connell’s (2005) masculinities schema are not fixed and/or defining of men’s lives. Usefully, objection, resignate, reimagined, and progress masculinities operationalize as process states in Connell’s (2005) masculinity theory. Methodologically, there is indeed value for secondary analyses of case studies to purposefully share men’s diverse illness vulnerabilities and resilience practices. Moreover, extending the reach of men’s stories affords additional and ongoing therapeutic benefits for those who read these rich empirical accounts.

There are of course numerous study limitations. Secondary analyses of data collected across more than 20 years from studies that did not primarily focus on illness vulnerabilities and resilience limit the findings. As Ruggiano and Perry (2019) note, re-analyzing data collected during another time period risks misrepresenting the social, cultural, and/or political norms for contextualizing and interpreting what participants said. That these case-study data were harvested from cross-sectional research also limits what can be said about each of the four participants over time. Some of these limitations can be addressed by conducting longitudinal research with a primary focus on men’s illness vulnerabilities and resilience.

To conclude, we should collectively resist silencing or stereotyping men’s illness vulnerabilities and resilience disclosures. Inevitably, vulnerabilities encroach on all men’s lives, and dialogue regarding the varied experiences and process states should be engaged as necessary and normative. While public testimonials and private qualitative interviews have been insightful, there are significant benefits to building cultural norms that listen and respond to men’s illness vulnerabilities and resilience through equity, diversity, and inclusion frames.
